# Modelling the feed intake response of growing pigs to diets contaminated with mycotoxins

**DOI:** 10.1017/S175173112000083X

**Published:** 2020-08

**Authors:** H. Nguyen-Ba, M. Taghipoor, J. van Milgen

**Affiliations:** 1 PEGASE, INRAE, Institut Agro, 35590 Saint-Gilles, France; 2 Faculty of Animal Science, Vietnam National University of Agriculture, Hanoi, Vietnam; 3 Université Paris-Saclay, INRAE, AgroParisTech, UMR Modélisation Systémique Appliquée aux Ruminants, 75005 Paris, France

**Keywords:** deoxynivalenol, resistance, resilience, quantification, individual variation

## Abstract

Quantifying robustness of farm animals is essential before it can be implemented in breeding and management strategies. A generic modelling and data analysis procedure was developed to quantify the feed intake response of growing pigs to perturbations in terms of resistance and resilience. The objective of this study was to apply this procedure to quantify these traits in 155 pigs from an experiment where they received diets with or without cereals contaminated with the mycotoxin deoxynivalenol (**DON**). The experimental pigs were divided equally in a control group and three DON-challenged groups. Pigs in each of the challenged groups received a diet contaminated with DON for 7 days early on (from 113 to 119 days of age), later on (from 134 to 140 days of age) or in both periods of the experiment. Results showed that the target feed intake trajectory of each pig could be estimated independently of the challenge. The procedure also estimated relatively accurately the times when DON was given to each challenged group. Results of the quantification of the feed intake response indicated that age and previous exposure to DON have an effect on the resilience capacity of the animals. The correlation between resistance and resilience traits was modest, indicating that these are different elements of robustness. The feed intake analysis procedure proved its capacity to detect and quantify the response of animals to perturbations, and the resulting response traits can potentially be used in breeding strategies.

## Implications

The feed intake analysis procedure shows its capacity to detect and quantify the feed intake response of growing pigs to a known perturbation and characterise these as resistance and resilience traits. These traits can be a potential source for genetic selection to breed animals for enhanced robustness.

## Introduction

Improving the capacity of animals to function well under a wide range of environmental conditions (i.e., robustness) has been of great interest in livestock production, especially through genetic selection (Knap, 2005). A prerequisite for selection is the quantification of the traits of interest. However, robustness is difficult to quantify because it consists of ‘dynamic elements such as the rates of response to, and recovery from, environmental perturbations’ (Friggens *et al.*, 2017). Because of these dynamic aspects, single time point measurements are not enough to quantify robustness. The limitation of single time point measurements can be illustrated by an example of two animals with different response mechanisms facing the same perturbation (adapted from Doeschl-Wilson *et al.* (2012)). Animal A is less affected by the perturbation than animal B (i.e., animal A is more resistant than animal B) but, once the perturbation is over, animal B recovers faster than animal A (i.e., animal B is more resilient than animal A). Thus, measuring the response of the animal at different stages of the perturbation will quantify different elements of robustness and can therefore have an impact on the breeding programme. This emphasises that the impact of a perturbation varies over time and results from different mechanisms of the response. Longitudinal data are therefore required to measure the dynamic response to and recovery from environmental perturbations (Friggens *et al.*, 2017).

With the development of monitoring technologies, production traits (e.g., feed intake and BW) can now be recorded at the individual level and with a very high frequency. Information extracted from this type of data has shown to be useful to characterise individual animal resilience (Putz *et al.*, 2018). Doeschl-Wilson *et al.* (2012) indicated that mathematical modelling offers the possibility to summarise complex mechanisms into a few parameters, thereby facilitating the ranking of animals. We recently developed a data analysis and modelling procedure to quantify the feed intake response of growing pigs to environmental perturbations in terms of resistance and resilience (Nguyen-Ba *et al.*, 2020). The procedure uses feed intake as input and deals with perturbations of known or unknown origin that decrease feed intake in pigs.

Mycotoxins are metabolites produced by fungi that can grow on cereals such as corn. The consumption of a mycotoxin-contaminated feed can result in reductions in feed intake and growth (Dersjant-Li *et al.*, 2003). Among the trichothecene mycotoxins, deoxynivalenol (**DON)** can have a profound effect on pigs due to their limited metabolic capacity to detoxify DON (Wu *et al.*, 2010). Although the consequences of DON on pigs have been well documented, the dynamic feed intake response during and after acute DON challenges has not been studied until recently. Serviento *et al.* (2018) studied the effects of DON on feed intake in individual growing pigs in relation to age and repeated exposure to DON. These data offer an opportunity to challenge and evaluate the novel procedure developed by Nguyen-Ba *et al.* (2020) to quantify resistance and resilience traits of growing pigs through their feed intake response to DON-contaminated diets and to compare these traits among animals.

## Material and methods

### Animals and treatments

Data from an experiment about the impact of DON on the feed intake of growing pigs were used (Serviento *et al.*, 2018). In brief, 155 growing pigs with an initial BW of approximately 50 kg were used in an experiment that lasted from 99 to 154 days of age. Pigs were distributed equally into four groups: a control group (**CC**) and three DON-challenged groups (**DC**, **CD** and **DD**). Pigs from group CC received a normal finishing diet throughout the study. Pigs from challenged groups also received the normal diet except during the challenge periods. The challenge periods lasted 7 days each during which pigs received a DON-contaminated diet. Pigs from group DC received a DON-contaminated diet early on in the experiment (i.e., from 113 to 119 days of age) and pigs from group CD received the DON-contaminated diet later on in the experiment (i.e., from 134 to 140 days of age). Pigs from group DD received the DON-contaminated diet during both aforementioned periods. All pigs were kept in the same room throughout the experiment. During the challenge periods, challenged and non-challenged pigs were kept in two separated zones to avoid cross-contaminations by faeces and urine. Feed was provided *ad libitum* during the experiment. Feed intake of individual pigs was recorded by automatic feeding stations and was computed on a daily basis.

### Description of the procedure to quantify resistance and resilience

A data analysis and modelling procedure was developed to quantify the feed intake response of growing pigs to perturbations in terms of resistance and resilience (Nguyen-Ba *et al.*, 2020). The procedure encompasses detection of perturbations and quantification of the pig’s response to a perturbation. The cumulative feed intake (**CFI**) rather than the daily feed intake (**DFI**) is used to detect perturbations in this procedure. The detection of perturbations is based on the hypothesis that there is a target trajectory curve of CFI (**target CFI**) that a pig desires to consume in a non-perturbed state. As a result of a perturbing factor, the CFI of the pig will deviate from the target CFI. Once the perturbing factor is over, the pig will try to regain its target CFI through compensatory feed intake. In the data analysis procedure, the target CFI was estimated by repeatedly fitting a polynomial function to CFI data in combination with the (temporary) elimination of data that lie below the fitted curve (i.e., observations that possibly result from the perturbed period). Then, a B-spline regression was used to fit a polynomial function to deviations from the target CFI. Information extracted from the B-spline function was used to estimate the start of each deviation and the time at which the difference between the CFI and the target CFI reached a maximum value. This maximum deviation corresponds to the point at which the pig starts to recover from a perturbation to regain its target CFI.

The second step quantifies the animal’s response to a perturbation. It is hypothesised that a perturbing factor has an immediate and constant negative effect on DFI that can be characterised by three parameters: t_start, t_stop and k1. The first two parameters indicate the time when a perturbation starts and ends, whereas k1 refers to the immediate and constant reduction in DFI at the start of the perturbation. The reduction in DFI will cause the CFI to deviate from the target CFI. The ratio between the CFI and the target CFI (**Ratio(t)**) is used as the driving force for a resilience mechanism that limits the negative effect of the perturbing factor. As soon as the perturbing factor is over, its negative effect on DFI disappears, but the resilience mechanism remains active. This results in compensatory feed intake that allows the CFI to approach the target CFI. The resilience and compensatory feed intake capacity of the animal are characterised by the parameter k2.

### Modifications of the procedure

Some aspects of the original procedure of Nguyen-Ba *et al.* (2020) were modified to make it (more) suitable for the data in this study. To estimate the target CFI in the original procedure, an autocorrelation test was combined with the temporal removal of data with negative residuals from the dataset. Since the duration of the experimental period is short (55 days), the procedure often stopped because of a criterion in the procedure to keep a minimum number of remaining observations. In the modified procedure, only the 10% quantile of data with negative residuals were removed at each filtration step, resulting in a more gradual estimation of the target CFI. Results of both methods are compared in Supplementary Material Table S1.

**Table 1:** Estimated model parameters of the target trajectory of cumulative feed intake of pigs that received a diet with or without deoxynivalenol (DON)-contaminated cereals

	Experimental group				RSE	*P*-value
	CC (*n* = 39)	DC (*n* = 39)	CD (*n* = 38)	DD (*n* = 39)		
Model parameters						
t_0_ (days)^1^	99.2	98.5	98.4	97.9	3.56	0.47
CFI_mid-point_ (kg)^1^	71.2	73.3	68.3	69.4	11.0	0.32
CFI_last_ (kg)^1^	156	159	150	150	19.2	0.11
Observed and calculated ADFI						
Observed ADFI (kg/d)^2^	2.87	2.79	2.67	2.59		<0.01
Target ADFI (kg/d)^2^	2.86	2.87	2.70	2.70	0.40	0.09

CC = group of pigs that received a non-contaminated control diet; DC = group of pigs that received a diet contaminated with DON from 113 to 119 days of age; CD = group of pigs that received a diet contaminated with DON from 134 to 140 days of age; DD = group of pigs that received a diet contaminated with DON in both aforementioned periods; RSE = residual standard error.

1 Parameter estimates of a polynomial model describing the target cumulative feed intake (CFI): t_0_ = age at which CFI equals 0; CFI_mid-point_ = CFI at the midpoint of the growing period; CFI_last_ = CFI at the last observation. See Nguyen-Ba *et al.* (2020) for details.

2 Average daily feed intake (ADFI) during the experiment (i.e., from 100 to 154 days of age): Observed ADFI = reported by Serviento *et al.* (2018); Target ADFI = calculated from the estimated model parameters.

In the original procedure, a perturbation was defined as a deviation of the CFI from the target CFI by at least 5% and for a duration of at least 5 days. To test the capacity of the B-spline function to identify the period(s) of distribution of the DON-contaminated diet, these criteria were not applied here. Any period during which the CFI deviated from the target CFI was characterised by the start time, the magnitude of the deviation and the duration (i.e., the time required for the CFI to regain the target CFI).

**Figure 1:**
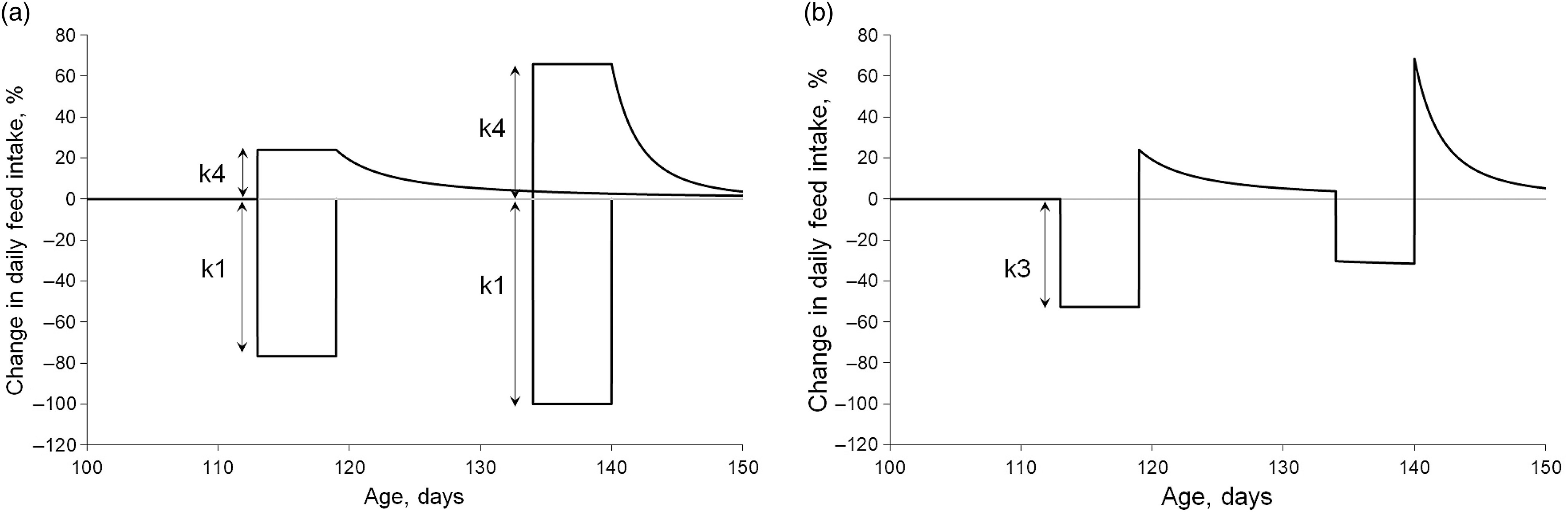
Change in daily feed intake (DFI) of a pig in reponse to receiving a diet contaminated with deoxynivalenol (DON) in two periods. Black lines = response mechanisms. The target DFI corresponds to no change in daily feed intake (grey line). Values smaller than 0 indicate the effect of resistance mechanisms and values greater than 0 indicate the effect of resilience mechanisms. The left panel (a) illustrates the response mechanisms where the reduction in DFI during the perturbation (k1) is counteracted for by a resilience mechanism (k2, the proportional change in DFI relative to the ratio between the actual cumulative feed intake and the target cumulative feed intake), which results in an attempt for compensatory feed intake (k4). The right panel (b) illustrates the actual change in DFI. The k3 corresponds to the difference between k1 and k4. During the second perturbation, the actual DFI is the result of the constant resistance and resilience mechanisms of the second perturbation and the declining resilience mechanism of the first perturbation.

The Ratio(t) defines the intensity of the resilience mechanism, which varies with time. For example, the CFI will be small at an early stage of life and a small reduction in DFI will result in a considerable reduction in Ratio(t). At later stages of life, the CFI will be much larger and the same perturbation will have little impact on Ratio(t). Ignoring the time dependency of Ratio(t) will lead to a biased estimation of k2. The original procedure was therefore modified to calculate Ratio(t) between CFI and the target CFI since the onset of the perturbation, and not since the start of the measurements. Equation (1) shows the modified Ratio(t) between CFI and the target CFI:(1)
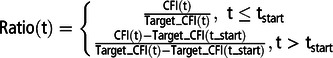
where ‘t’ is the age of the animal (days of age), ‘t_start’ is the time (days of age) when the perturbation starts, ‘CFI(t)’ and ‘Target_CFI(t)’ are the CFI and the target CFI (kg) at day ‘t’, respectively, and ‘Target_CFI(t_start)’ is the target CFI at day ‘t_start’. The shape of the response of the resilience mechanism is different in the modified procedure compared to the original procedure. In the original procedure, the resilience capacity of the pig during the perturbation becomes progressively bigger (grey curve of figure 2 in Nguyen-Ba *et al.*, 2020), whereas in the modified procedure the resilience capacity causes a constant increase in DFI during the perturbation (Figure 1).

**Figure 2:**
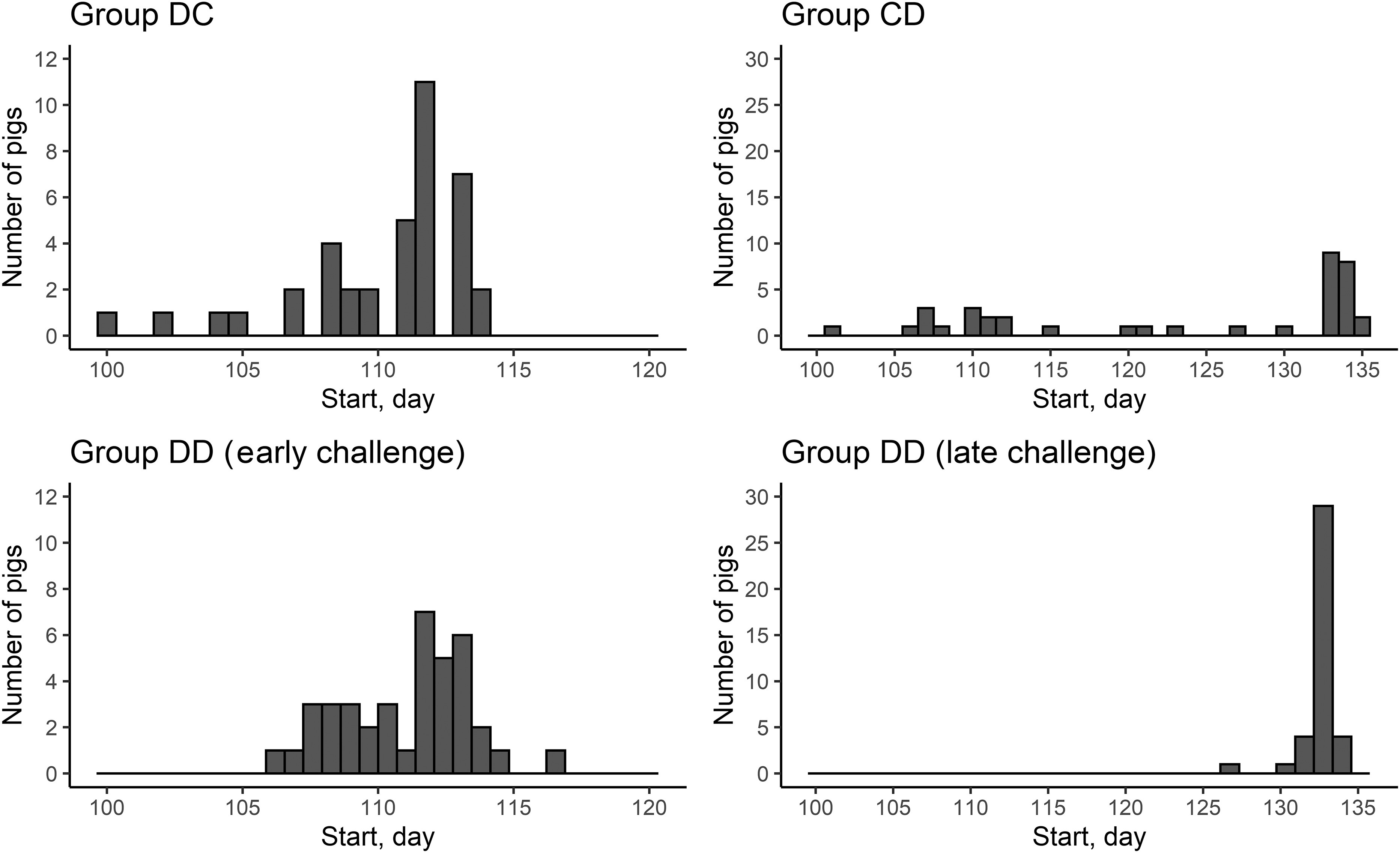
Capacity of the data analysis procedure to identify the start of the distribution of a diet contaminated with deoxynivalenol (DON) based on the feed response of pigs. In groups DC and DD (early challenge), pigs received the DON-contaminated diet from 113 days of age onwards. In groups CD and DD (late challenge), pigs received the DON-contaminated diet from 134 days of age onwards.

Pigs in the group DD received the DON challenge twice. Each perturbation was modelled with independent resistance and resilience mechanisms as shown in Figure 1. Equations are described as:(2a)
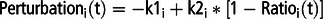

(2b)
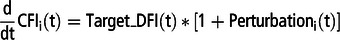
where ‘Perturbation_i_(t)’ is the dynamic change in CFI at time ‘t’ when the pig responds to a perturbation *i* (*i* = 1 and *i* = 2 correspond to the first and second DON challenge, respectively) and ‘Ratio_i_(t)’ represents the ratio associated with perturbation *i* as described in equation (1). Target_DFI(t) represents the target trajectory curve of DFI. Changes in DFI (relative to the target DFI) in the response to each DON challenge depend on the dynamic effects of ‘Perturbation_i_(t)’. The sum of the changes relative to the target DFI results in the actual DFI as:(3)
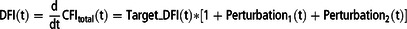



The value of Perturbation_i_(t) will be negative during the period of the perturbing factor and positive thereafter. The independence of the mechanisms of disturbance and recovery means that an animal may be recovering from the first challenge (through compensatory DFI) while, at the same time, it is affected by another challenge resulting in a reduction in DFI.

Because the change in DFI is constant during the period when the DON-contaminated diets are distributed, two other traits were estimated. The constant reduction in DFI during the DON challenge resulting from the resistance and resilience mechanisms can be calculated as k3 = k1/(1 + k2). Likewise, the instantaneous increase in DFI once the feeding of the DON-contaminated diet stops is given by k4 = k1 × k2/(1 + k2).

In the original procedure, all four parameters of the perturbation model (t_start, t_stop, k1 and k2) were estimated. With the current dataset, it appeared difficult to estimate all these parameters, because the period of distributing the DON-contaminated diet lasted only 7 days. For group DD, this would require the estimation of 11 parameters and, with 55 DFI observations, this can easily lead to an overparameterisation of the model. It was therefore decided to fix t_start and t_stop at the start and end times of the distribution of the DON-contaminated diets.

### Statistical analysis

Parameter estimates for the target CFI and results from the B-spline functions were compared among the four groups to evaluate the capacity of the procedure to detect perturbations. The quantification of the response to DON challenges was carried out in the three challenged groups (i.e., DC, CD and DD). Since the parameters t_start and t_stop were fixed, only the resistance and resilience parameters were estimated. The k3 is the constant reduction in DFI during the perturbation and is easier to interpret than k1. The model was therefore parameterised to estimate k2 and k3, and k1 and k4 were calculated from these parameter estimates.

All statistical and modelling procedures were performed using R software version 3.6.1 (http://cran.r-project.org/). The optimisation was performed by the non-linear function ‘nlsLM’ of the package ‘minpack.lm’. To characterise the pig’s response to a perturbation, equations (2b) and (3) were solved using the ‘ode’ function of the ‘desolve’ package with an integration step size (dt) of one day. The optimisation was done using the ‘optim’ function. Statistical comparison was carried out using a one-way ANOVA test (the R base function ‘aov’). Pearson correlations between parameters were calculated using the R base function ‘cor’. In all analyses, differences were considered as statistically significant if *P* < 0.05 and as tendencies if *P* < 0.1.

## Results

### Estimation of the target cumulative feed intake and detection of perturbations

The estimated parameters of the target CFI (t_0_, CFI_mid-point_ and CFI_last_) and the average daily feed intake (**ADFI**) were compared among the four groups (Table 1). No significant differences were found in the parameter estimates of the target CFI among the four groups.

**Figure 3:**
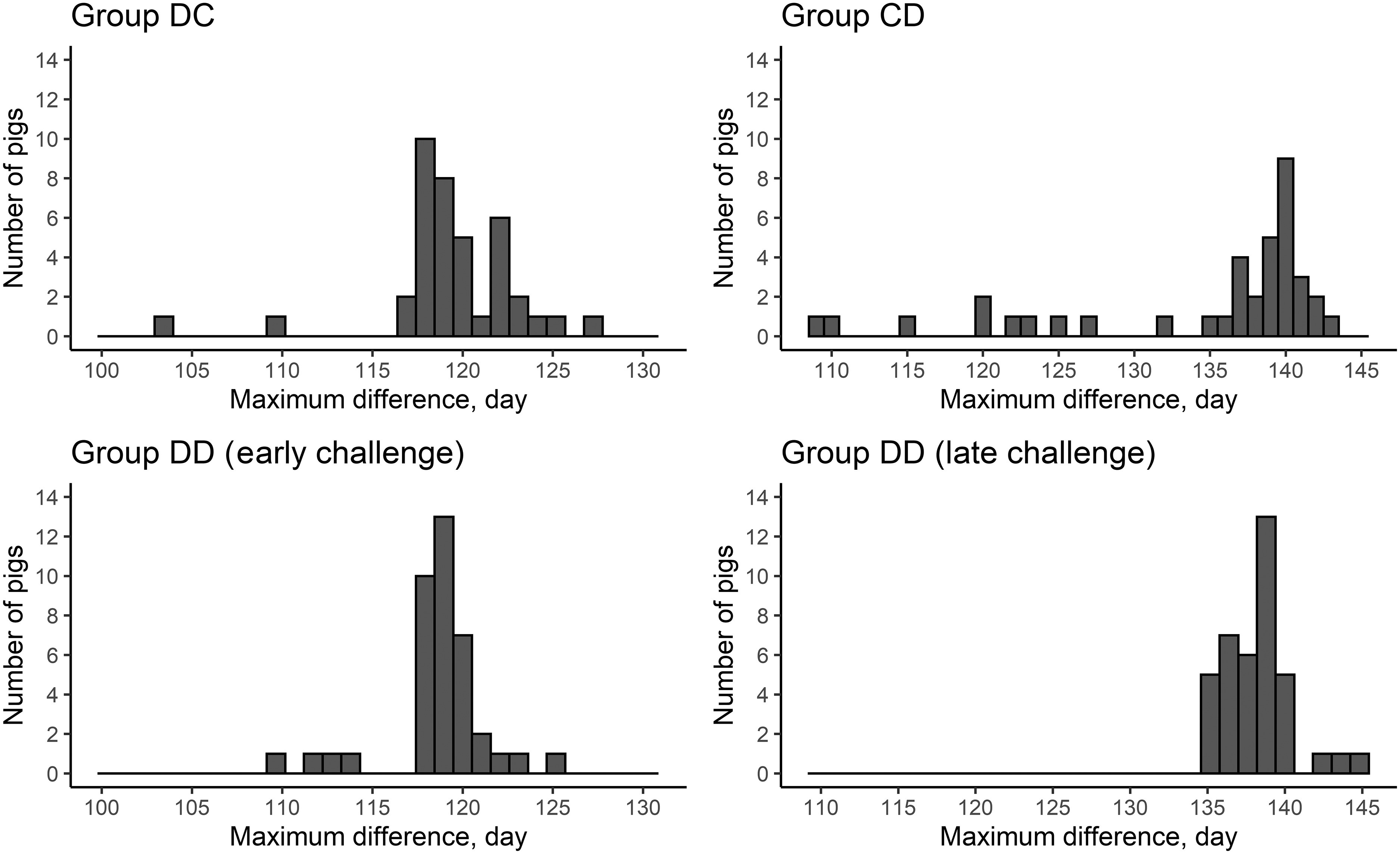
Capacity of the data analysis procedure to identify the day from which pigs started to recover after having received a diet contaminated with deoxynivalenol (DON). The procedure determines the day when the difference between the actual cumulative feed intake and the target cumulative feed intake is maximal. In groups DC and DD (Early challenge), pigs stopped receiving the DON-contaminated diet from 119 days of age onwards. In groups CD and DD (Late challenge), pigs stopped receiving the DON-contaminated diet from 140 days of age onwards.

In general, the procedure detected deviations in the CFI for all groups, even in group CC. However, the magnitude of the deviations in group CC was very small. Multiple deviations identified by the procedure indicate that the CFI of pigs was affected by factors other than by the DON-contaminated diet alone. As the objective of this study was to quantify the response of pigs to DON challenges, only the deviations related to DON-challenged periods were examined. The identified deviations are shown in Figures 2 and 3, respectively, for the start of the deviation and for the time at which the maximum difference between the CFI and the target CFI occurred. The procedure identified that most deviations started and reached the maximum value near the distribution of the DON-contaminated diet. However, there were cases where the starting time and the time of the maximum deviation between the CFI and the target CFI were before or after the distribution of the DON-contaminated diet, especially for group CD.

### Characterisation of the response of pigs to a diet contaminated with deoxynivalenol

For five pigs (four in group DD and one in group CD), the estimation procedure did not converge and data for these pigs were not considered further in the analysis.

The estimated model parameters are given in Table 2. Between the two groups receiving the DON-contaminated diet early on (i.e., group DC and the first perturbation of group DD), no significant differences were observed in the estimated values of k2 and k3. Between the two groups receiving the DON-contaminated diet later on (i.e., group CD and second perturbation of group DD), k3 was significantly lower and k2 significantly higher for pigs that received the DON challenge for the second time (group DD) compared to that of pigs that received this challenge for the first time (group CD).

**Table 2:** Model parameters for the resistance and resilience potential of pigs that received a diet contaminated with deoxynivalenol (DON) during one or two periods

		Experimental group				
		DC (*n* = 39)	CD (*n* = 37)	DD (*n* = 35)	RSE	*P*-value
Estimated model parameters^1^						
k3						
Early challenge		0.46		0.46	0.23	0.99
Late challenge			0.42	0.31	0.18	0.02
k2						
Early challenge		0.81		0.90	0.73	0.57
Late challenge			1.59	2.36	0.94	<0.001
Calculated model parameters^2^						
k1						
Early challenge		0.77		0.77	0.37	0.98
Late challenge			1.05	1.03	0.56	0.91
k4						
Early challenge		0.31		0.32	0.19	0.96
Late challenge			0.63	0.72	0.41	0.36

DC = group of pigs that received a diet contaminated with DON in the first challenge from 113 to 119 days of age; CD = group of pigs that received a diet contaminated with DON in the second challenge from 134 to 140 days of age; DD = group of pigs that received a diet contaminated with DON in both aforementioned periods; RSE = residual standard error.

1 k3 = net reduction in daily feed intake (DFI) during the DON challenge period relative to the target DFI; k2 = proportional change in DFI relative to the ratio between the actual cumulative feed intake (CFI) and the target CFI.

2 k1 = instantaneous reduction in DFI at the start of the DON challenge period relative to the target DFI; k4 = compensatory feed intake capacity over and above the target DFI.

The effect of age or BW can be assessed by comparing the results of pigs receiving the DON-contaminated diet early on (i.e., group DC and the early challenge of group DD) with those of pigs receiving the DON challenge for the first time later on in life (i.e., group CD). For k3, no difference was found between the early and late DON challenge (0.46 for groups DC and DD *v*. 0.42 for group CD; *P* = 0.39). However, pigs those challenged early in life had a significant lower value of k2 than those challenged later in life (0.85 for groups DC and DD *v*. 1.59 for group CD; *P* < 0.001).

The results for the calculated model parameters k1 and k4 are also given in Table 2. The k3 can be interpreted as the result of a negative effect on DFI (through k1) and a positive effect on DFI (through k4). The responses of k1 and k4 resembled those observed for k3 but were more variable. The values of k1 ranged from 10% to 346%, whereas those of k4 ranged from 0 to 246%.

**Figure 4:**
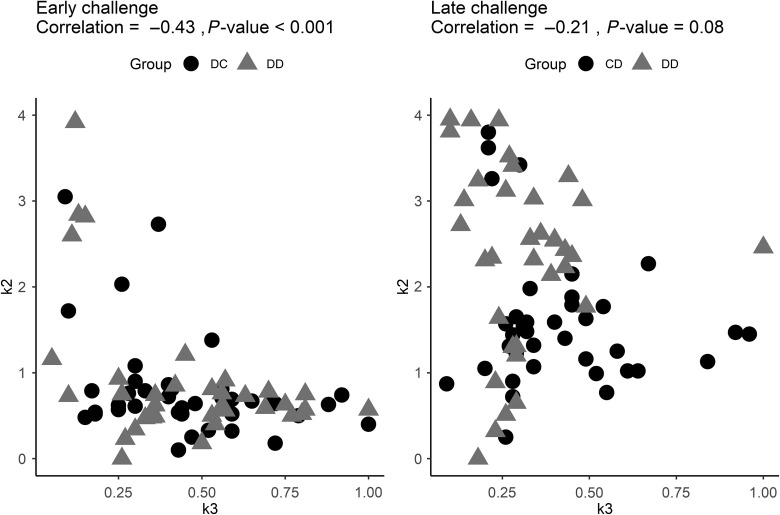
Correlations between the change in daily feed intake during the perturbation (k3) and the resilience capacity (k2) of pigs when receiving diets contaminated with deoxynivalenol (DON). The left panel denotes the periods when pigs received a DON-contaminated diet from 113 to 119 days of age (early challenge) and the right panel indicates when they received the DON-contaminated diet from 134 to 140 days of age (late challenge). DC = group of pigs that received a diet contaminated with DON from 113 to 119 days of age; CD = group of pigs that received a diet contaminated with DON from 134 to 140 days of age; DD = group of pigs that received a diet contaminated with DON in both aforementioned periods.

The correlations between k2 and k3 for the two DON challenge periods are given in Figure 4. The correlation was moderately negative for the early challenge period (*P* < 0.001) and only tended to differ from zero for the late challenge period (*P* = 0.08).

## Discussion

The capacity of an animal to minimise the effect of environmental perturbations and to quickly retrieve its pre-perturbed condition, usually termed resistance and resilience, are important elements in sustainable livestock production. The complex and dynamic nature of the mechanisms of animal’s response to a perturbation makes modelling a promising approach to propose and to quantify the underlying mechanisms. This study demonstrates that a modelling and data analysis procedure can be applied to characterise resistance and resilience traits of animals, allowing to identify variability among growing pigs in their feed intake response to a DON-contaminated diet.

### Estimation of the target cumulative feed intake

Determining the production potential of an animal is important for animal breeding but can be difficult to estimate because deviations of this potential can occur due to disturbances, resulting in the actually measured production trait (Berghof *et al.*, 2018). Differences among animals in average feed intake have been correlated to heritable health-related traits (Putz *et al.*, 2018). However, disturbances and the corresponding response of the animal may vary over time. Average performance traits are not suitable to capture the dynamic aspects of robustness, and they may even mask the underlying mechanisms of resistance and resilience. For example, a reduction in DFI may be followed by a period of full compensatory DFI and the average DFI of such an animal may not be different from a situation without a perturbation. On the other hand, an animal that is affected by a constant perturbation throughout its life will have an average production lower than its true potential without a perturbation (Berghof *et al.*, 2018).

The parameters of the target CFI curve did not differ between the four treatment groups resulting in similar values for the average target DFI (Table 1). This differs from the results of Serviento *et al.* (2018) who reported differences in the observed ADFI among the groups. Moreover, the average target DFI of the control group CC was very similar to the observed ADFI (Table 1). This suggests that the procedure is capable to extract a target trajectory from the actual data. The numerically lower average target DFI values for treatments CD and DD may indicate that the procedure was not completely successful, but this may also be due to the relatively short recovery period for these late-challenged animals (14 days), which may have been too short to regain the target CFI.

This study is based on the hypothesis that the animal has a target to attain. The CFI was used as a target rather than the DFI because it is easier to envisage a target for a state variable (i.e., kg of feed) than for a rate variable (kg of feed/day). The notion of a target is also represented in growth models such as the logistic or Gompertz functions, in which the growth rate is a function of the target mature BW. Revilla *et al.* (2019) used this approach to model the response of piglets around weaning. They used a Gompertz function (as a target) in combination with a perturbation model to represent changes in BW after weaning. These changes were modelled through a possible reduction in BW immediately followed by a recovery phase to regain the trajectory of the Gompertz function.

The assumption of the existence of a target trajectory that the animal seeks to attain is debatable. There may be situations in which the animal responds to a perturbation but where it will not (or cannot) seek to regain the target trajectory. A classic example of this is the study of Lister and McCance (1967) who restricted feed intake in piglets so that they maintained their BW at 5.5 kg for 1 year. When feed was offered *ad libitum* after 1 year, the previously restricted pigs had initially the same growth rate as those that were not restricted, but stopped growing at the same chronological age as the non-restricted pigs. This indicates that restricted pigs could not reach the same target mature BW as the control group (or that they had changed their target mature BW).

### Characterisation of the feed intake response

The start time and time required for the maximum deviation estimated by the procedure corresponded reasonably well to the actual start and the end of DON challenge. The relatively short challenge period of 7 days in combination with a perturbation model with potentially four parameters could lead to difficulties to estimate the model parameters and it was therefore decided to fix the t_start and t_tstop at the actual distribution times of the DON-contaminated diet. Although these time points were close to the corresponding parameters estimated by the procedure, there were cases where the procedure indicated that the perturbation started before the distribution of the DON-contaminated diet (especially for the CD and DD groups). This may be due to another unidentified perturbing factor not related to the experiment.

Fixing the start and end times of perturbation to the times during which the DON-contaminated diet was distributed does not allow to estimate a ‘lag time’ during which the animal is exposed but does not respond to the perturbing factor (Sandberg *et al*, 2006). Likewise, the approach used here does not allow to have a ‘remnant’ perturbing effect by which the animal responds to but it is no longer exposed to the perturbing factor. The consequence of our approach is that the response of the animal is characterised by only two parameters (k3 and k2). But the estimates of these parameters may be somewhat biased in cases where the start and end times of the response do not correspond to the distribution times of the DON-contaminated diet. The structure of the perturbation model requires sufficient data to estimate all four parameters, and DFI data for 7 days is not sufficient to realise this. It is possible that exploring the feed intake behaviour and meal patterns provide additional information on the response of the animal to the perturbation, but this requires a different model structure, which is beyond the scope of this paper.

The ratio between the actual CFI and the target CFI (i.e., Ratio(t)) is used as a driving force for the resilience mechanism in the model. As indicated before, we have changed the time point from whereon Ratio(t) was determined. This change has consequences on the simulated response during the perturbation, but it also has implications on the interpretation of the model in terms of resistance and resilience. The k1 is seen as the immediate and constant response to a perturbation (i.e., resistance), which is counteracted by the resilience parameter k2. In the original approach, this resulted in that the reduction in DFI during the perturbation gradually diminished because of the two mechanisms. This gradual decrease was provoked by the time dependency of Ratio(t) that also caused that estimates of k2 became time-dependent. This issue was solved by determining Ratio(t) from the start of each perturbation, which results in a constant reduction in DFI during the perturbing period (k3). The k3 is thus to be interpreted as the result of a constant resistance mechanism (k1) and a constant resilience mechanism (k4). The k4 also reflects the degree of compensatory feed intake over and above the target DFI at t_tstop. Although k3 is to some extent ‘observable’ in DFI data, this is not the case for k1. There were a number of cases where both k1 and k4 exceeded 100%, with values of k3 in the 0% to 100% range. Values greater than 100% for k1 are difficult to interpret biologically because this would mean that the animal tries to have a negative DFI. Values greater than 100% are possible for k4 if the animal is capable to double its DFI during compensatory feed intake. Rather than interpreting k3 in terms of k1 and k4, k3 can also be interpreted by itself, implying that resistance is the only mechanism during the perturbation and that resilience only starts once the perturbation is over (through k2 or k4).

The constant reduction in DFI during feeding the DON-contaminated diet differs from the average observations of the change in DFI of Serviento *et al.* (2018). They observed an important reduction in DFI during the first day of the challenge, and this reduction became progressively less important to surpass the DFI of the control group resulting in compensatory DFI (see Figure 4 of Serviento *et al.*, 2018). There are two explanations for this difference. Firstly, in the modelling procedure used here, the CFI was used as a response criterion, and changes in CFI may be less sensitive than changes in DFI. Secondly, it is possible that the current model does not fully correspond to the dynamics of the response of the animal and should be adapted so that the effect of a perturbing factor diminishes with time of exposure. As indicated by Nguyen-Ba *et al.* (2020), different aspects of the data analysis procedure can be adapted, as was done here for the determination of Ratio(t). A diminishing effect of the perturbing factor could have been considered but may not be sufficient to accurately describe the change in DFI because as described above, the DON challenge may cause a remnant effect on the animal. As indicated in Figure 4 of Serviento *et al.* (2018), once feeding the DON-contaminated diet stopped, the DFI of the challenged animals was close to or slightly below of that of the control group. Compensatory DFI occurred only a few days after feeding the normal uncontaminated diet. Modelling the response during and after feeding the DON-contaminated diet would therefore probably require more than the two (or four) parameters considered in the current study.

### Between-group differences

Parameters estimated from the model confirmed findings of the experiment (Serviento *et al.*, 2018) that the response of pigs to DON-contaminated diet is influenced by age or BW and by a previous exposure to the DON-contaminated diet. Interestingly, the older pigs recovered faster than the younger pigs from the DON challenge (k2 averaged 0.85 for group DC and the early challenge of group DD *v*. 1.59 for group CD). Pigs that had received the DON-contaminated diet early in life were less affected by receiving this diet again later on compared with those that received it for the first time and they also tended to recover faster (i.e., smaller k3 and greater k2 for the second challenge of pigs of group DD compared to those of group CD).

The degree of compensatory DFI once the normal diet is fed again (i.e., k4) was greater for the late-challenged pigs (when they are also older and heavier) compared to the early challenged pigs. Gut capacity is often assumed to be a limiting factor for feed intake in young pigs and gut capacity increases with BW (Nyachoti *et al.*, 2004). The initial BW for the early challenged pigs was 72 kg, whereas it was 94 kg for the late challenged pigs. The greater compensatory DFI for the heavier pigs is therefore in line with the idea that gut capacity becomes less of a limiting factor with increasing BW. However, the difference between the two groups is considerable, implying that the heavier pigs could increase their DFI by more than 60% over their target DFI, compared to ‘only’ 30% for the lighter pigs.

From the effects of both age and repeated exposure to DON, it is tempting to speculate that the adaptation of pigs relies more on resilience than on resistance mechanisms. The immune system is one of the major targets of mycotoxins (Pierron *et al.*, 2016). Depending on the dose, exposure frequency and animal species, mycotoxins can have either immunostimulatory or immunosuppressive effects (Bondy and Pestka, 2000). Exposure to a high dose of DON has been reported to reduce the cellular and humoural immune responses, thereby decreasing the host resistance to infectious diseases (Pestka *et al.*, 2004; Oswald *et al.*, 2005). In pigs, ingestion of DON with the doses close to that used in Serviento *et al.* (2018) caused a depression in the immune response against the porcine reproductive and respiratory syndrome virus (Savard *et al.*, 2014) and inhibition of the vaccine efficiency (Savard *et al.*, 2015).

### Individual variability in the response of pigs to the deoxynivalenol-contaminated diet

Considerable variation among pigs in their response to a DON-contaminated diet was observed in this study (Figure 4). Bishop and Morris (2007) reported genetic variation in the response of sheep and goats to different types of mycotoxins. Breeding against mycotoxin susceptibility may be feasible due to a moderate to high heritability of phenotypic measurements (Bishop and Morris, 2007). Therefore, the findings of our study provide opportunities to consider resistance and resilience traits to select pigs for coping with mycotoxins. Moreover, resistance and resilience to DON seem to be independent traits as only low and moderate correlations between k2 and k3 were found in two DON challenge periods.

## Conclusion

This study showed the possibility to apply the model proposed by Nguyen-Ba *et al.* (2020) to situations where the origin of the perturbation is known. The procedure detected deviations from the target CFI resulting from the distribution of a DON-contaminated diet. A previous exposure to a DON-contaminated diet alleviated the decrease in DFI following a second exposure. Older and heavier pigs seem to be more resilient than younger and lighter pigs. The low to moderate correlations between the resistance and resilience traits suggest these are different elements of robustness.

## Software and data repository resources

The data used in the study were provided by Serviento *et al.* (2018). The data analysis procedure is a modified version of the procedure Nguyen-Ba *et al.* (2020), which can be obtained from doi.org:10.1017/S1751731119001976. The modified version of the R code can be obtained from the authors upon request.

## Supplementary material

For supplementary material accompanying this paper visit https://doi.org/10.1017/S0033291718004130. click here to view supplementary material
