# Supplementary material for: Modelling the feed intake response of growing pigs to diets contaminated with mycotoxins
Source: Animal. 2020 Apr 30;14(Suppl 2):s303–12. doi: 10.1017/S175173112000083X (PMC7391214; doi:10.1017/S175173112000083X)
Supplement: Supplementary file 1 [file S175173112000083Xsup001.pdf]

***Animal* journal**

Modelling the feed intake response of growing pigs to diets contaminated with mycotoxins

H. Nguyen-Ba[1, 2], M. Taghipoor[3] and J. van Milgen[1]

[1] *PEGASE, INRAE, Institut Agro, 35590 Saint-Gilles, France*

[2] *Faculty of Animal Science, Vietnam National University of Agriculture, Hanoi, Vietnam*

[3] *Université Paris-Saclay, INRAE, AgroParisTech, UMR Modélisation Systémique Appliquée aux Ruminants, 75005, Paris, France*

Corresponding author: Jaap van Milgen. Email: jaap.vanmilgen@inrae.fr

Supplementary material

In the original procedure of Nguyen-Ba *et al*. (2019), the target trajectory of cumulative feed intake was obtained by repeatedly fitting a polynomial function to the cumulative feed intake data. If a significant autocorrelation was observed among the residuals, data associated with negative residuals were temporarily eliminated from the dataset, and the fitting procedure was repeated. The procedure was terminated when the autocorrelation among the residuals was no longer significant. It was also ensured that at least 20 data points remained, irrespective of if there was autocorrelation among the residuals.

With the original procedure, if there is autocorrelation among the residuals, roughly half of the data is eliminated at each step of the fitting procedure. This procedure may be too severe for the current data set, which is composed of only 55 data points for each individual pig. In the modified procedure, only the 10% quantile of data with negative residuals were eliminated at each step of the fitting procedure. The results of both procedures are compared in Table S1.

Table S1 *Comparison of procedures to estimate the target trajectory curve of cumulative feed intake in growing pigs*

	Original procedure[1] (n = 155)	Modified procedure[2] (n = 155)
Average number of filtration steps	2.0 ± 0.2	6.3 ± 1.2
The fitting procedure stopped because there was no longer autocorrelation among the residuals:		
Number of animals concerned	39	78
Number of data points used to estimate the target cumulative feed intake	27.7 ± 3.6	29.3 ± 6.3
The fitting procedure stopped to ensure that there were at least 20 data points remaining, but autocorrelation remained among the residuals:		
Number of animals concerned	116	77
Number of data points used to estimate the target cumulative feed intake	28.7 ± 3.0	23 ± 0.0

[1] Original procedure: the procedure to estimate the target cumulative feed intake was done by eliminating all data with negative residuals at each filtration step.
[2] Modified procedure: the procedure to estimate the target cumulative feed intake was done by eliminating the 10% quantile of data with negative residuals at each filtration step.

Reference

Nguyen-Ba H, Van Milgen J and Taghipoor M 2020. A procedure to quantify the feed intake response of growing pigs to perturbations. Animal 14, 253-260.
